# Postoperative 30-day complications after cemented/hybrid versus cementless total hip arthroplasty in osteoarthritis patients > 70 years: A multicenter study from the Lundbeck Foundation Centre for Fast-track Hip and Knee replacement database and the Danish Hip Arthroplasty Register

**DOI:** 10.1080/17453674.2020.1745420

**Published:** 2020-04-14

**Authors:** Martin Lindberg-Larsen, Pelle Baggesgaard Petersen, Christoffer Calov Jørgensen, Søren Overgaard, Henrik Kehlet, Lundbeck Foundation Center for Fast-track Hip and Knee Arthroplasty Collaborating Group

**Affiliations:** a Lundbeck Foundation Centre for Fast-track Hip and Knee Arthroplasty;; b Orthopaedic Research Unit, Department of Orthopaedic Surgery and Traumatology, Odense University Hospital, Department of Clinical Research, University of Southern Denmark;; c Section for Surgical Pathophysiology, Rigshospitalet, Copenhagen, Denmark

## Abstract

Background and purpose — The use of cementless total hip arthroplasty (THA) in elderly patients is debated because of increased risk of early periprosthetic femoral fractures. However, cemented femoral components carry a risk of bone cement implantation syndrome. Hence, we compared in-hospital complications, complications leading to readmission and mortality ≤ 30 days postoperatively between hybrid/cemented (cemented femoral component) vs. cementless THA in osteoarthritis patients > 70 years.

Patients and methods — This is a prospective observational cohort study in 9 centers from January 2010 to August 2017. We used 30-day follow-up from the Danish National Patient Registry, patient records, and data from the Danish Hip Arthroplasty Register. Only THAs performed as a result of osteoarthritis were included.

Results — 3,368 (42%) of the THAs were cemented/hybrid and 4,728 (58%) cementless. The in-hospital complication risk was 7.7% after cemented/hybrid vs. 5.3% after cementless THA (< 0.001), statistically not significant when adjusting for comorbidities (p = 0.1). There were similar risks of complications causing readmission (5.7% vs. 6.2%) and mortality ≤ 30 days (0.2% vs. 0.3%). 15 cases (0.4%) of pulmonary embolism (PE) were found after cemented/hybrid vs. 4 (0.1%) after cementless THA (p = 0.001); none occurred within 24 hours postoperatively. 2 of the PEs after cementless THA led to mortality. Cemented/hybrid THA remained significantly associated with risk of PE (RR 3.9, p = 0.02), when adjusting for comorbidities. BMI > 35 was associated with highest risk of PE (RR 5.7, p = 0.003). The risk of periprosthetic femoral fracture was 0.2% after cemented/hybrid vs. 1.5% after cementless THA (p < 0.001) and the risk of dislocations was 1.2% after cemented/hybrid THA vs. 1.8% after cementless THA (p = 0.04).

Interpretation — The higher risk of PE after cemented/hybrid THA and higher risk of periprosthetic femoral fractures and dislocations after cementless THA highlights that both medically and surgically complications are related to fixation technique and have to be considered.

The use of a cementless fixation technique in total hip arthroplasties (THA) is generally preferred compared with a cemented technique in patients with hip osteoarthrosis and age < 70 years in most Western countries. The use of cementless implants in THA has increased in all age groups including the elderly (AOANJRR 2018, DHR 2018, NJR 2018) despite the fact that cementless femoral components are associated with increased risk of early periprosthetic femoral fractures, especially in patients > 70 years (Makela et al. 2014, Thien et al. 2014, Lindberg-Larsen et al. 2017, Tanzer et al. 2018).

A concern regarding the use of cemented femoral components is the risk of the so-called bone cement implantation syndrome. The syndrome occurs at the time of bone cementation and prosthesis insertion where the high intra-medullary pressure forces medullary fat emboli into the circulation resulting in risk of pulmonary embolism (Orsini et al. 1987, Donaldson et al. 2009, Segerstad et al. 2019). The clinical presentation of this syndrome may range from hypoxia to fulminatory pulmonary and systemic marrow embolization, right ventricular failure, and circulatory collapse (Issack et al. 2009). The incidence has been difficult to determine due to the variation of severity of the syndrome. In a series of 1,016 cemented hemiarthroplasty patients, the incidence of the most severe grade of the syndrome (grade 3) was 1.7% and associated with mortality of 88%. Risk factors for the syndrome were ASA grade III–IV, chronic obstructive pulmonary disease, and medication with diuretics or warfarin (Olsen et al. 2014). However, the exact incidence and consequences of bone cement implantation syndrome after total hip arthroplasty remains unknown. As occurrence of severe bone cement implantation syndrome would likely influence postoperative length of hospital stay, readmission risk, or mortality, we compared in-hospital complications, complications leading to readmission, and mortality within 30 days postoperatively between hybrid/cemented (cemented femoral component) versus cementless THA in osteoarthritis patients > 70 years.

## Patients and methods

**Figure 1.**
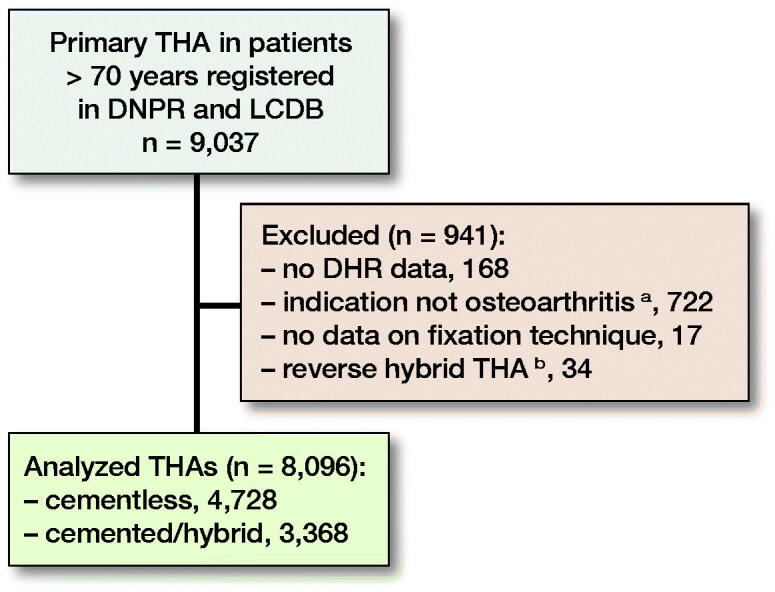
Flow-chart inclusion. DNPR: Danish National Patient Register; LCDB: Lundbeck Foundation Centre for Fast-track THA and TKA Database; DHR: Danish Hip Arthroplasty Register. **^a^**Including fracture, rheumatoid arthritis, tumor cases, and sequelae after fracture. **^b^**Cemented acetabular component and cementless femoral component.

This is a prospective observational cohort study on patients from the Lundbeck Foundation Centre for Fast-track THA and TKA database (LCDB 2019). The STROBE guidelines for reporting observational studies were followed. Data on 9,037 primary unselected elective THA procedures performed in patients > 70 years were prospectively collected between February 2010 and August 2017 in 9 departments reporting to the LCDB. Supplementary data on fixation technique, duration of surgery, indication, and previous surgeries were available from the Danish Hip Arthroplasty Registry (DHR 2018) in 98% of cases. Hence, 8,096 primary elective THA procedures performed in patients with primary osteoarthritis were available for analysis after exclusions (Figure 1). The indication for either cemented/hybrid or cementless THA was based on surgeon and center preference and varied between centers. The percentage of cemented/hybrid THA ranged from 4% to 95% with great interdepartmental variation.

The procedures were divided into cemented/hybrid (cemented femoral components) versus cementless THA (cementless femoral and acetabular components). Reverse hybrid THA procedures (cementless femoral component and cemented acetabular component) were excluded. Patients registered in the DHR as operated on for indications other than osteoarthritis were excluded. Furthermore, patients having simultaneous bilateral procedures or an additional major arthroplasty procedure within 90 days were excluded (Figure 1).

### The Lundbeck Foundation Centre for Fast-track THA and TKA Database (LCDB)

The LCDB is a prospective database on unselected consecutive primary hip and knee arthroplasty procedures from 9 contributing centers initiated in January 2010 in 6 Danish centers, and an additional 3 centers joined the collaboration in 2012, 2013, and 2014 (LCDB 2019). The LCDB contains data on preoperative comorbidity and patient characteristics such as pharmacologically treated cardiopulmonary disease, diabetes, previous venous thromboembolism (VTE), alcohol consumption, smoking habits, and living conditions (Table 1). Data are prospectively collected from patients within 1 month before surgery using self-completed questionnaires with staff available for assistance. Validation of the consistency of the preoperative patient questionnaire has been performed previously using matched patient medical records (Pitter et al. 2016).

**Table 1.** Patient characteristics. Values are number (%) unless otherwise specified

Patient characteristics	Cemented/ hybrid THA ^a^ n = 3,368 (42%)	Cementless THA n = 4,728 (58%)
Mean age (range)	79 (71–100)	76 (71–97)
Female sex	2,311 (69)	2,641 (56)
Mean BMI (range)	26 (14–64)	26 (15–61)
missing	24 (0.7)	29 (0.6)
Use of walking aid	1,374 (41)	1,421 (30)
missing	78 (2.3)	98 (2.1)
Smoking	341 (10)	448 (9.5)
missing	23 (0.7)	27 (0.6)
Alcohol > 2 units/day	114 (3.4)	375 (7.9)
missing	36 (1.1)	26 (0.5)
Type 1 diabetes	25 (0.7)	24 (0.5)
missing	22 (0.7)	25 (0.5)
Type 2 diabetes	319 (9.5)	463 (9.8)
missing	20 (0.6)	11 (0.2)
Pharmacologically treated cardiovascular disease	664 (20)	831 (18)
missing	42 (1.2)	40 (0.8)
Pharmacologically treated pulmonary disease	362 (11)	421 (8.9)
missing	24 (0.7)	26 (0.5)
Prior cerebral stroke	219 (6.5)	350 (7.4)
missing	65 (1.9)	60 (1.3)
History of VTE	261 (7.7)	367 (7.8)
missing	95 (2.8)	60 (1.3)
Hereditary VTE	362 (11)	470 (9.9)
missing	347 (10)	623 (13)
Hypercholesterolemia	1,422 (42)	1,780 (38)
missing	25 (0.7)	24 (0.5)
Anticoagulative treatment	689 (21)	850 (18)
missing	32 (1.0)	49 (1.0)
Preoperative anemia ^b^	1,193 (35)	1,313 (28)
missing	23 (0.7)	34 (0.7)
Hypertension	1,994 (59)	2,689 (57)
missing	11 (0.3)	20 (0.4)
Pharmacologically treated psychiatric disorder	259 (7.7)	303 (6.4)
missing	28 (0.8)	23 (0.5)

**^a^**Hybrid = cemented femoral component

**^b^** Hb < 13 g/dL.

THA: total hip arthroplasty

VTE: venous thromboembolism

The study period for the current updated detailed analysis was from January 2010 until August 2017. All collaborating centers adhered to similar fast-track protocols, including use of spinal anesthesia (≈90%), multi-modal opioid-sparing analgesia, early mobilization, and discharge to own home based on functional discharge criteria. Thromboprophylaxis was prescribed according to local guidelines including thromboprophylaxis administered 6–8 hours after surgery, consisting of either rivaroxaban (Xarelto, Bayer Pharma, Berlin, Germany) 10 mg/day, enoxaparin (Klexane, Sanofi-Aventis, Paris, France) 4,000 IU/day, dalteparin (Fragmin, Pfizer Health Care, New York, USA) 5,000 IU/day, or fondaparinux (Arixtra, GlaxoSmithKline, London, UK) 2.5 mg/day. Thromboprophylaxis was used only during hospitalization if LOS was ≤ 5 day. If LOS > 5 days recommendations on duration varied in the study period. From 2010 to 2016 international guidelines with thromboprophylaxis for up to 35 days for THA were recommended and from 2016 the Danish recommendation changed to 6–10 days (RADS 2016). Compression stockings or intermittent pneumatic compression devices were not used. Pulsatile lavage was used as a standard procedure in cemented THA surgery in the participating centers.

### Outcomes

Complications within 30 days postoperatively leading to length of hospital stay (LOS) > 4 days, readmission, and mortality were analyzed. All patients with a LOS of > 4 days had their medical records examined to determine the reason for prolonged LOS and in-hospital complications during primary admission. Using the patients’ unique Danish social security numbers, we obtained information on LOS, 30-day readmissions, and mortality from the Danish National Patient Registry. As reporting to the Danish National Patient Registry is mandatory for hospitals to receive reimbursement, an almost complete follow-up (> 99%) is assured (Schmidt et al. 2015). All unplanned readmissions with an overnight hospital stay within 30 days postoperatively were evaluated to determine postoperative complications after discharge. Discharge records or patient records of readmitted patients were scrutinized and relation to index surgery was evaluated. Causes of mortality within 30 days were evaluated using patient records.

In the case of pulmonary embolism (PE) in the discharge summary the entire medical record was evaluated to confirm the occurrence of PE and identify any possible causative events. The PE diagnosis was in all cases confirmed by spiral computed tomography (CT).

#### Statistics

Overall comparative analysis of the groups was performed using Student’s t-test for normally distributed data and crude comparisons of proportions were done using a chi-square test. Analysis of potential risk factors associated with in-hospital complications and PE was performed using a multivariable Poisson regression model with robust error variance (Zou 2004). Cases with missing data were excluded from analysis. A sensitivity analysis on risk of PE, periprosthetic fractures, and dislocations after excluding 165 (85 + 80) cemented/hybrid THA from 2 centers performing < 10% cemented/hybrid THA and 14 cementless THA from 1 center performing < 10% cementless THA was performed.

Results are given as relative risk (RR) estimates or percentages with 95% confidence intervals (CI). Any p-value of < 0.05 was considered significant. Analysis was done using SPSS version 22 (IBM Corp, Armonk, NY, USA).

### Ethics, registration, funding, and potential conflicts of interests

According to Danish law no approval from the regional ethics committee was required as the study was non-interventional. Permission to obtain and store data without informed consent was obtained from the Danish Patient Safety Authority (3-3013-56/2/EMJO) and the Danish Data Protection Agency (RH-2014-132). The LCDB is registered as an ongoing study registry on clinicaltrials.gov (NCT01515670). This study was funded by the Lundbeck Foundation Centre for Fast-Track Hip and Knee Arthroplasty, Copenhagen, Denmark. No competing interests were declared.

## Results

8,096 primary elective THA procedures performed in 7,786 osteoarthritis patients > 70 years were analyzed (Figure 1). In 42% of the procedures a cemented or hybrid (cemented femoral component) fixation technique was used and 58% were performed using a cementless technique. There were more females in the cemented/hybrid group (69%) compared with the cementless group (56%), but otherwise demographics were similar between groups (Table 1).

**Figure 2.**
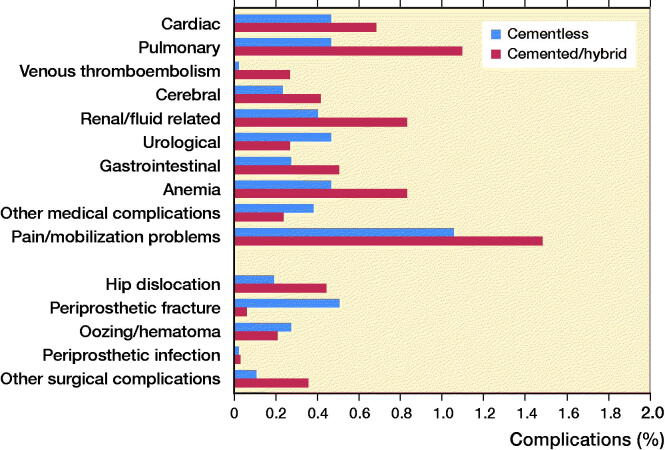
In-hospital complications causing LOS > 4 days.

The in-hospital complication rate was 7.7% after cemented/hybrid THA vs. 5.3% after cementless THA (< 0.001) (Table 2), but statistically not significant (RR 1.1, p = 0.2) when adjusting for preoperative comorbidity (Table 3). The most frequent in-hospital complication causing prolonged LOS > 4 days was pain and mobilization problems after both cemented/hybrid and cementless THA (1.5%, n = 50 and 1.1%, n = 50 respectively). All in-hospital complications are presented in Figure 2.

**Table 3.** Multivariable Poisson regression analysis of potential risk factors influencing the risk of in-hospital complications

Potential risk factor	RR (95% CI)
Cemented/hybrid THA	1.1 (0.9–1.3)
Pharmacologically treated cardiovascular disease	1.5 (1.2–1.8)
Pharmacologically treated pulmonary disease	1.4 (1.1–1.8)
History of venous thromboembolism	1.6 (1.2–2.0)
BMI ≥ 35	2.1 (1.5–2.9)
Use of mobility aid	2.1 (1.8–2.6)
Age (per 10 years)	2.1 (1.8–2.5)

RR: Relative risk; BMI: body mass index.

**Table 2.** Postoperative 30-day outcome. Values are count (%)

Outcomes	Cemented/ hybrid THA n = 3,368	Cementless THA n = 4,728	p-value
Mortality	8 (0.2)	15 (0.3)	0.5
In-hospital complications	260 (7.7)	252 (5.3)	< 0.001
Complications causing readmission	193 (5.7)	292 (6.2)	0.4
Pulmonary embolism	15 (0.4)	4 (0.1)	0.001
Periprosthetic femoral fractures	7 (0.2)	70 (1.5)	< 0.001
Dislocations	42 (1.2)	87 (1.8)	0.04

**Figure 3.**
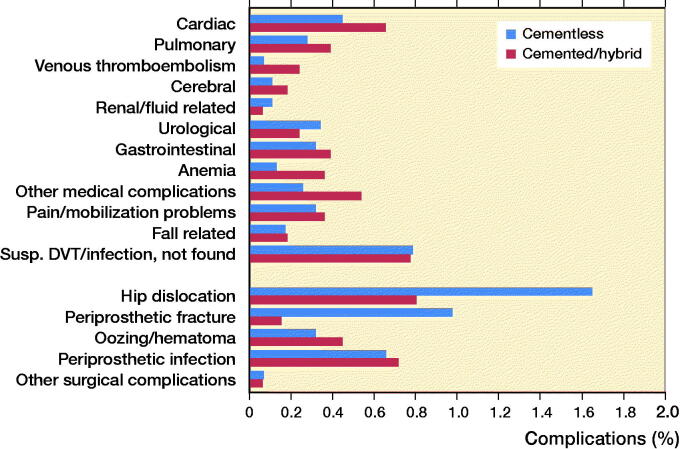
Complications causing readmission ≤ 30 days postoperatively.

The 30-day overall readmission risk was 6.5% after cemented/hybrid vs. 7.0% after cementless THA. When subtracting readmissions due to suspected but disproven VTE/infections the readmission risk was 5.7% after cemented/hybrid vs. 6.2% after cementless THA (p = 0.4). The most frequent complications causing readmission were hip dislocations (0.8%, n = 27), periprosthetic joint infection (0.7%, n = 24), and cardiac complications (0.7%, n = 22) after cemented/hybrid THA whereas hip dislocations (1.6%, n = 78) and periprosthetic femoral fractures (1.0%, n = 46) were most frequent after cementless THA (Figure 3).

7 cases of PE during primary admission and 8 PEs leading to readmission ≤ 30 days were found after cemented/hybrid THA compared with 2 cases of PE during primary admission and 2 PE leading to readmission after cementless THA. Pulmonary embolism (PE) and time course from surgery as well as information on thromboprophylaxis at time of PE are presented in Table 4. 2 of the PEs after cementless THA led to mortality. Hence, 15 cases (0.4%) of PE were found after cemented/hybrid vs. 4 (0.1%) after cementless THA within 30 days postoperatively (p = 0.001). None of the PEs occurred within 24 hours postoperatively. Cemented/hybrid THA remained significantly associated (RR 3.9, p = 0.022) with risk of PE in the regression model (Table 5).

**Table 5.** Multivariable Poisson regression analysis of potential risk factors influencing the risk of pulmonary embolism (PE)

Potential risk factor	RR (95% CI)
Cemented and hybrid THA	3.9 (1.2–13)
Pharmacologically treated cardiovascular disease	1.9 (0.7–5.3)
Pharmacologically treated pulmonary disease	1.0 (0.2–4.8)
History of venous thromboembolism	2.0 (0.6–6.6)
BMI ≥ 35	5.7 (1.6–20)
Use of mobility aid	1.6 (0.6–4.4)
Age (per 10 years)	1.6 (0.6–4.2)

**Table 4.** Pulmonary embolism (PE) and time course from surgery

Days from surgery to PE	Place of PE diagnosis	Ongoing thrombo- prophylaxis	Previous VTE	Preoperative anticoagulant therapy ^a^	Age	PE-related mortality
Cemented/hybrid (n = 15)						
2	Primary admission	Yes	Yes		85	
2	Primary admission	Yes			75	
2	Primary admission	Yes		Yes	86	
2	Primary admission	Yes			81	
3	Readmission	No			77	
3	Primary admission	Yes			78	
4	Primary admission	Yes	Yes		82	
7	Readmission	No			84	
8	Readmission	No			92	
10	Readmission	No			73	
13	Readmission	No			71	
14	Readmission	No			87	
23	Primary admission	Yes	Yes		79	
26	Readmission	Yes		Yes	84	
27	Readmission	No			84	
Cementless (n = 4)						
1	Primary admission	Yes			73	
2	Readmission	Yes		Yes	78	Yes
14	Primary admission	Yes			71	Yes
17	Readmission	No			89	

**^a^**All 3 patients treated with warfarin pre- and postoperatively.

VTE: venous thromboembolism.

2 cases of periprosthetic femoral fractures during primary admission and 5 leading to readmission ≤ 30 days were found after cemented/hybrid THA compared with 24 periprosthetic femoral fractures during primary admission and 46 leading to readmission after cementless THA (Figures 2–3). Hence, 7 cases (0.2%) of periprosthetic femoral fractures were found after cemented/hybrid vs. 70 (1.5%) after cementless THA within 30 days postoperatively (p < 0.001). In 1 of 7 cases of periprosthetic fractures after cemented/hybrid THA revision surgery was needed and the remaining 6 cases were treated nonoperatively. In contrast, 39 of 70 cases of periprosthetic fracture after cementless THA needed revision surgery and the remainder were treated nonoperatively (25 cases) or treated at primary surgery with wiring/osteosynthesis (6 cases).

The risk of dislocations was 1.2% (15 during primary admission and 27 causing readmission) after cemented/hybrid THA vs. 1.8% (9 during primary admission and 78 causing readmission) after cementless THA (p = 0.04).

A sub-analysis on risk of dislocations comparing groups based on acetabular fixation was performed and the risk of dislocations was similar: 1.6% (5 during primary admission and 3 causing readmission) after the use of cemented vs. 1.6% (19 during primary admission and 102 causing readmission) after cementless acetabular components.

The postoperative mortality ≤ 30 days was 0.2% after cemented/hybrid vs. 0.3% after cementless THA (p = 0.5). Time course and causes of 30-day mortality are presented in Table 6.

**Table 6.** Mortality, time course from surgery and cause of death

Days from surgery to death	Place of death	Age	Cause
Cemented/hybrid THA (n = 8)			
2	Primary admission	86	Ileus
3	Primary admission	76	Respiratory insufficiency
4	Home	100	Cardiac arrest (unknown)
8	Primary admission	79	Stroke
13	Readmission	77	Myocardial infarction
22	Readmission	90	Renal insufficiency
25	Primary admission	79	Stroke
29	Readmission	88	Endocarditis
Cementless THA (n = 15)			
2	Readmission	78	Pulmonary embolism
2	Readmission	71	Cardiac arrest
3	Primary admission	78	COPD, sepsis, ARI
5	Readmission	82	UTI, sepsis
5	Home	82	Cardiac arrest (arrhythmia)
6	Primary admission	79	Pneumonia
8	Primary admission	87	Stroke
13	Readmission	88	Stroke
13	Home	74	Unknown
14	Readmission	71	Pulmonary embolism
14	Primary admission	87	Pneumonia
14	Primary admission	86	Myocardial infarctionI
18	Readmission	71	Unknown
23	Readmission	90	Pneumonia
23	Readmission	94	Pneumonia

COPD: chronic obstructive pulmonary disease;

ARI: acute renal insufficiency;

UTI: urinary tract infection

A sensitivity analysis on risk of PE, periprosthetic fractures, and dislocations after excluding 165 (85 + 80) cemented/hybrid THA from 2 centers performing < 10% cemented/hybrid THA and 14 cementless THA from 1 center performing < 10% cementless THA revealed no differences from the risk estimates presented above.

## Discussion

The use of cementless THA implants in patients > 70 years is debatable due to the increased risk of early periprosthetic fractures (Makela et al. 2014, Lindberg-Larsen et al. 2017, Tanzer et al. 2018). One of the arguments for the use of cementless implants may be the risk of bone cement implantation syndrome (Olsen et al. 2014) and the potentially associated mortality after cementation (McMinn et al. 2012, Garland et al. 2017). However, the actual impact of the use of cement on the overall risk of postoperative complications has not been described in detail previously. In this study, we report a higher risk of in-hospital complications after cemented/hybrid THA (cemented femoral component) compared with cementless THA (7.7% vs. 5.3%); however, this was unrelated to fixation technique when adjusting for preoperative patient comorbidities. Furthermore, an increased risk of 30-days postoperative PE after cemented/hybrid compared with cementless THA was found, and also when adjusting for comorbidities. The higher risk of PE can hardly be directly explained by cementation as none happened on the day of surgery or the first day after and it seems unlikely that a potential low-grade bone cement implantation syndrome during surgery can affect the cardiopulmonary system, making the patient more at risk of PE later. The lack of a direct and timely relationship between cement implantation and the occurrence of postoperative PE suggests that other factors, such as obesity and delayed mobilization, may also contribute (Table 4). Furthermore, many of the PE events happened despite ongoing thromboprophylaxis, indicating that these events happen in high-risk patients, requiring further investigations as discussed in detail by Petersen et al. (2019). We therefore feel that the term “bone cement implantation syndrome” should be used with more caution in this context.

Whether the patients suffering PE in our study had detectable bone cement implantation syndrome intraoperatively cannot be confirmed in this study. Hence, our finding of clinically documented postoperative PE of 0.4% after cemented/hybrid THA is significantly higher than after cementless THA, but still much lower compared with the paraclinically reported incidence (28% [all grades]) of bone cement implantation syndrome (Olsen et al. 2014).

Postoperative mortality in relation to the use of a cemented fixation technique in THA has been analyzed on a larger scale using data from the Swedish Hip Arthroplasty Register (n = 178,784), finding a minimally increased relative risk of early mortality < 15 days postoperatively after cemented/hybrid THA compared with a matched control group from the general population, but the relative risk of mortality reversed > 15 days postoperatively (Garland et al. 2017). We found similar mortality rates between groups, but a larger cohort is needed to detect potentially statistically significant differences in mortality.

The higher risk of periprosthetic fractures after cementless THA, especially in the elderly (Makela et al. 2014, Thien et al. 2014, Lindberg-Larsen et al. 2017), is confirmed in our study, amounting to 1.5% after cementless THA compared with 0.2% after cemented/hybrid THA. Periprosthetic fractures may cause protracted mobilization with limited weight-bearing and when revision surgery is needed the procedures are complex and often associated with a high risk of complications and mortality (Bhattacharyya et al. 2007, Griffiths et al. 2013). Our study includes only 30 days’ follow-up and hence more periprosthetic fractures, especially after cementless THA, with associated patient morbidity may be recognized later (Lindberg-Larsen et al. 2017).

The higher risk of postoperative dislocations after cementless THA compared with a cemented/hybrid technique is in line with the higher revision rates due to dislocations reported in the Danish Hip Arthroplasty Register over the past decades after a cementless fixation technique for the femoral and acetabular components (DHR 2018). A higher risk of dislocations after cementless THA has previously been explained by more variation in the positioning of the cementless acetabular component (Chawda et al. 2009). However, we found that the risk of dislocation was similar (1.6%) when comparing cementless versus cemented acetabular components in our cohort in contrast to previous findings, supporting that the risk of dislocation is multifactorial.

The high proportion of cementless THA procedures (58%) performed in our population > 70 years is surprising, but in line with 53% cementless THAs performed in Denmark in 2017 overall in the same age group (DHR 2018). This finding confirms the overall Danish trend of using cementless THA in all age groups.

A limitation of this study is confounding by indication as no similar indication criteria for the use of a cemented fixation technique existed between centers. We tried to limit this by performing a sensitivity analysis on risk of PE, periprosthetic fractures, and dislocations after excluding cemented/hybrid THA from 2 centers performing < 10% cemented/hybrid THA and cementless THA from 1 center performing < 10% cementless THA. However, the results from the sensitivity analysis did not differ from the main analysis.

Although the registered preoperative patient comorbidity was similar, other confounding factors such functional status and bone quality (Dorr type) (Nash and Harris 2014) might have influenced surgeons’ choice. The type of cementless femoral component used may also have influenced the risk of periprosthetic fractures (Gromov et al. 2017) and this was not analyzed in the present study. Furthermore, the few PE events might lead to overfitting of the multivariable Poisson risk-factor analysis, potentially introducing a “type II error.” Another limitation of the study is the observational nature that excludes the possibility to report on causality. However, our study provides detailed information on complications leading to prolonged LOS or readmissions after cemented/hybrid THA versus cementless THA, which is necessary for shared decision-making between surgeons and patients.

A strength of this study includes prospective recording of numerous baseline characteristics, thus minimizing recall bias, an unselected consecutive patient population, and follow-up through a high-quality nationwide register, thereby ensuring data completeness (Schmidt et al. 2015). Other strengths are the standardized multicenter fast-track setup, and the relatively short and recent study period. Furthermore, not relying on only diagnostic codes but using review of discharge notes and patient records for specific causes of morbidity ensures > 99% follow-up for somatic readmissions (Schmidt et al. 2015) and eliminates the dependency on the questionable reliability of a discharge diagnosis within the DNPR (Severinsen et al. 2010, Sundboll et al. 2016, Bedard et al. 2018).

In conclusion, we report a higher risk of PE after cemented/hybrid THA, but the risk of periprosthetic femoral fractures and dislocations was higher after cementless THA. These findings highlight that, both medically and surgically, complications are related to fixation technique and have to be considered in the decision-making on an individual level.
